# Knowledge and Willingness for Organ Donation in the Middle Eastern Region: A Meta-analysis

**DOI:** 10.1007/s10943-019-00883-x

**Published:** 2019-07-15

**Authors:** Ahammed Mekkodathil, Ayman El-Menyar, Brijesh Sathian, Rajvir Singh, Hassan Al-Thani

**Affiliations:** 1 Department of Surgery, Clinical Research, Trauma Surgery, Hamad Medical Corporation (HMC), Doha, Qatar; 2 Department of Surgery, Trauma and Vascular Surgery, Clinical Research, Hamad General Hospital, HMC, P.O Box 3050, Doha, Qatar; 3 Clinical Medicine, Weill Cornell Medical College, Doha, Qatar; 4 Biostatistics, Cardiology Research Center, Heart Hospital, HMC, Doha, Qatar; 5 Department of Surgery, Trauma and Vascular Surgery, HMC, Doha, Qatar

**Keywords:** Organ donation, Willingness, Knowledge, Middle East

## Abstract

Medical advancements over the past decades brought organ transplantation as a definitive therapy for different end-stage organ failure. However, non-availability of organs required for transplantation is a major challenge worldwide. We aimed to determine the knowledge and willingness to donate organs in various populations and settings in the Middle Eastern region. Literature searches were conducted on PubMed, MEDLINE, Cochrane, and Google scholar electronic databases. Different combinations of search terms such as “organ donation”; “knowledge”, “awareness”; “beliefs”, “willingness”; and “attitude” along with the country names were used. Additional searches using reference lists of studies and review articles were conducted. Data were extracted using standardized excel form and pilot tested. Three authors independently abstracted the data using a data collection form. Results from different studies were pooled for the analysis when appropriate. The search yielded 1806 articles; 1000 duplicates and review articles were excluded, and a further 792 articles not relevant were excluded. Finally, 14 original studies met the inclusion criteria. Total pooled sample size for assessing knowledge was 6697 and for willingness was 8714. Pooled overall knowledge regarding organ donation was 69% with a 95% CI [64.5, 73.5]. Pooled overall willingness to donate organ was 49.8% with a 95% CI [41.3, 58.4]. Knowledge about organ donation and willingness to donate organs varies in different population and settings in the Middle East. These in fact are linked to multiple social factors ultimately leads to ‘consent’ for donating organs by a potential donor. Family’s influence; religious, traditional and spiritual beliefs; and status of ethnic, minority, and immigrant populace are the important determinants of the decision for organ donation. Understandings on social determinants in organ donation remain crucial and should be addressed while developing policies and organizational developments.

**Electronic supplementary material:**

The online version of this article (10.1007/s10943-019-00883-x) contains supplementary material, which is available to authorized users.

## Introduction

Organ donation is a global scenario enabling transplantation of organs, cells, and tissues which are recognized as the optimal treatment for end-stage organ diseases (Shaheen and Souqiyyeh 2004). First successful living and deceased kidney transplantations took place in Boston in 1954 and 1962, respectively, led by Dr. Joseph Murray and Dr. David Hume. The 1960s and 1970s witnessed more solid organ transplantation around the world, like lung, liver, pancreas, and heart transplantation. The 1980s marked critical medical achievement against organ rejection through the appropriate use of immune suppression medications. Better tolerance toward organ transplantation was achieved by the end of the last century (LiveOnNY (UNOS) 2015). This very brief history suggests that the medical entity of organ transplantation has been attaining more accountability in clinical grounds.

However, evidence suggests that the demand of organs for transplantation is increasing constantly (Matesanz et al. 2009; GODT (WHO) 2013). Recently, the Global Observatory on Donation and Transplantation (GODT) showed that solid organ transplantations which are conducted each year worldwide (approximately 110,000) is less than 10% of the actual demand (GODT (WHO) 2013).

The GODT data also revealed the disparity existing between the developed industrialized Western countries and developing Asian and African countries in terms of organ donation and transplantation (GODT (WHO) 2013). These disparities are not only limited to economy or technology, but also exists in specific interventions adopted, kind of organizations involved, legal and policy-related dynamics. The GODT aimed at an international coherence by introducing a global database which also includes information about legislature and organizational activities of different member states of the World Health Organization (WHO).The GODT database includes 109 WHO member states which in fact covers more than 85% of the global population. Notably, 74% of these countries do have an official body responsible for overseeing and coordinating donation and transplantation activities and 80% have specific legislation for organ procurement and transplantation (Matesanz et al. 2009; GODT (WHO) 2013). The waiting list for organ transplantation across different nations where specific organ procurement system is existing shows a definite gap between the supply and demand of organs, and this gap is found to increase constantly (Shaheen and Souqiyyeh 2004; Matesanz et al. 2009; Caplan et al. 2009). The issue is not solely because of unavailability of organ donors but due to the low rates of conversions of potential donors into actual donors.

The aim of this meta-analysis was to determine the knowledge and willingness toward organ donation in various populations and settings in the Middle East.

## Methods

This meta-analysis was conducted and reported according to the Preferred Reporting Items for Systematic Reviews and Meta-Analyses (PRISMA) Statement [Supplementary Table].

### Literature Searches

Literature searches were conducted on PubMed, MEDLINE, Cochrane library, and Google scholar electronic databases. The search terms used include “organ donation”; “knowledge”, “awareness”; “beliefs”, “willingness” and “attitude” in various combinations in the title or abstract AND different country names listed in the Middle Eastern region. Additional searches were conducted using reference lists of studies and review articles for selection of relevant articles.

### Inclusion/Exclusion Criteria

The inclusion criteria were (1) original studies, (2) English language; (3) published in the period from 01 January 2005 through 31st January 2018; (4) assessed willingness to donate organs; (5) patient population was from the Middle East listed countries; and (6) patients of any age, gender, and ethnicity. Articles other than original studies such as reviews, letters to the editor and commentaries were excluded. The Middle East region includes Bahrain, Cyprus, Egypt, Iran, Iraq, Israel, Jordan, Kuwait, Lebanon, Oman, Palestine, Qatar, Saudi Arabia, Syria, Turkey, United Arab Emirates, and Yemen (Özalp 2011).

The consensus on inclusion/exclusion criteria was reached based on the fact that whether the study provides information about knowledge or willingness to donate organs in the listed countries in the Middle Eastern region, regardless of the type of study population. Therefore, studies with small sample sizes were also included. Only studies available with full texts were included and abstracts without full texts were excluded.

### Mesh Terms

MeSH terms employed during search process include “organ procurement”, “knowledge, attitudes, practice”, “awareness”, “culture”, and “religion”.

### Data Extraction

The titles of the studies resulted from the database searches were screened initially and relevant papers were selected. Then the abstracts and full texts were reviewed according to the inclusion criteria for final selection. The titles, abstracts and full-text articles were reviewed independently by three researchers (AE, AM & BS). Extracted data included authors, origin of studies, source population, study setting and period, inclusion/exclusion criteria, data sources and measurement, sample size, age/gender distribution, awareness, and willingness to donate.

### Methodological Quality

The methodological quality of the selected studies was assessed based on five STROBE criteria from checklist such as study design, setting, participants, data sources/measurement, and study size. The STROBE checklist and the five criteria selected from the checklist were most relevant in the assessment of methodological quality of observational studies in epidemiology.

### Data Analysis and Synthesis

Descriptive statistics and 95% confidence interval were used to summarize willingness percentage estimated from individual studies. The decision to select either fixed effect or random effects model depends on results of statistical tests for heterogeneity. Data heterogeneity was assessed using the Cochrane *Q* homogeneity test with significance set at *p* < 0.10. If the studies were statistically homogeneous, fixed effect model was selected. A random effects model was used when studies were statistically heterogeneous. The *I*^2^ test is the ratio of true heterogeneity to the total variation in observed effects. A rough guide to interpretation of *I*^2^ test is 0 to 25%: might not be important; 25 to 50%: may represent moderate heterogeneity; 50 to 75%: may represent substantial heterogeneity; and more than 75%: considerable heterogeneity.

Pooled estimates were calculated using R 3.5.1 software.

## Results

**Fig. 1:**
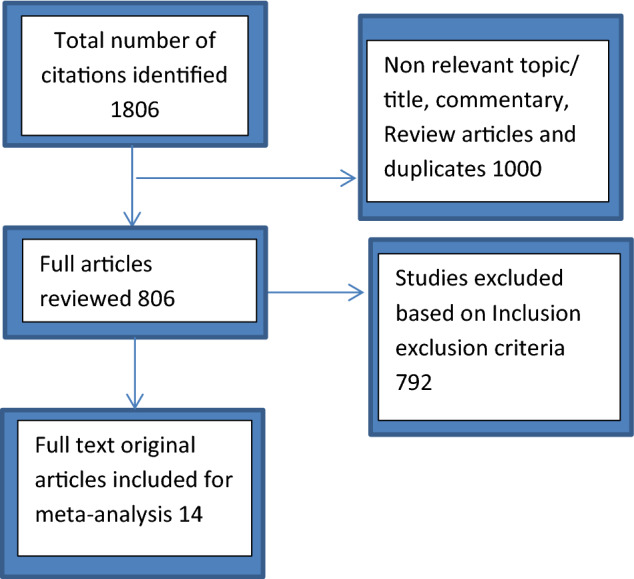
Flow diagram of study selection process for systematic review

The PubMed, Medline, Cochrane library, Google scholar, and reference list search generated 1806 articles; 1000 duplicates and review articles were excluded; relevant titles and/or abstracts underwent detailed evaluation, and a further 792 articles were further eliminated from the analysis leaving finally 14 original studies that met all inclusion criteria (Fig. 1, Table 1).

**Table 1:** Description studies included in the review of literature

Author (year)	Origin	Study design and population	Sampling	Results/conclusion	STROBE
Abbasi et al. (2018)	Iran	Cross-sectional survey; medical students	Convenience sampling (*n* = 165)	Willingness to donate: (49.69%)	Complete
Sayedalamin et al. (2017)	Saudi Arabia	Cross-sectional survey	Convenience sampling (*n* = 481)	Knowledge (90%)	Complete
		Medical students		Willingness to donate 41.2%	
Agrawal et al. (2017)	Saudi Arabia	Cross-sectional survey	Convenience Sampling (*n* = 403)	Knowledge (44.7%)	Complete
		Patients attending outpatient department		Willingness to donate (24.5%)	
Merdad et al. (2017)	Saudi Arabia	Cross-sectional survey; healthcare students	Convenience sampling (*n* = 597)	Knowledge (78%)	Complete
				Willingness to donate: (89%)	
Elsafi et al. (2017)	Saudi Arabia	Cross-sectional survey; allied health	Convenience sampling (*n* = 434)	Willingness to donate: (44%)	Complete
Al Habeeb et al. (2017)	Saudi Arabia	Cross-sectional general population	Convenience sampling (*n* = 1250)	Knowledge (91%)	Complete
				Willingness to donate 43.6%	
Flayou et al. (2016)	Morocco	Cross-sectional survey	Convenience sampling (*n* = 245)	Knowledge (36.3%)	Complete
		Nursing and medical students		Willingness to donate (65.7%)	
Al Bshabshe et al. (2016)	Saudi Arabia	Cross-sectional survey	Convenience sampling (*n* = 873)	Knowledge (93%)	Complete
		University students		Willingness to donate 76.2%	
Almohsen et al. (2016)	Saudi Arabia	Cross-sectional survey	Convenience sampling (*n* = 195)	Knowledge (61.5%)	Complete
		University students		Willingness to donate 37.4%	
Afzal Aghaee et al. (2015)	Saudi Arabia	Cross-sectional survey; fresh man of various medical discipline	Convenience sampling (*n* = 400)	Knowledge (41.5%)	Complete
				Willingness to donate: (55.6%)	
Alsaied et al. (2012)	Qatar	Cross-sectional survey; Healthcare workers in ICU and ED	Cluster sampling (*n* = 418)	Willingness to donate: physicians (24%) and nurses (20%) were less favorable compared to technicians (44%)	Complete
Alam (2007)	Saudi Arabia	Cross-sectional survey; general population	Random sampling (*n* = 948)	Knowledge about organ donation (92%); Willingness to donate (42%)	Complete
Shahbazian et al. (2006)	Iran	Cross-sectional; survey general population	Cluster sampling (*n* = 1000)	Willingness to donate (64%)	Complete
El-Shoubaki and Bener (2005)	Qatar	Cross-sectional survey; general population (GP attenders)	Multistage cluster sampling (*n* = 1305)	Knowledge about organ donation (69%)	Complete
				Willingness to donate (37.8%)	

Table 1 shows that willingness to donate organs is related to knowledge about organ donation (Abbasi et al. 2018; Sayedalamin et al. 2017; Agrawal et al. 2017; Merdad et al. 2017; Elsafi et al. 2017; Al Habeeb et al. 2017; Flayou et al. 2016; Al Bshabshe et al. 2016; Almohsen et al. 2016; Afzal Aghaee et al. 2015; Alsaied et al. 2012; Alam 2007; Shahbazian et al. 2006; El-Shoubaki and Bener 2005). Among the 14 included studies, knowledge was measured by administering a questionnaire which included the questions addressing various aspects of organ donation. Most of these questions were true or false type. Generally knowledge on organ donation were addressed in the questionnaires as following: (1) general knowledge about the organ donation, (2) awareness on procurement and distribution of donated organs, (3) religious and cultural understanding on organ donation, and (4) awareness among ethnic minority population regarding the relevance of organ donation (Abbasi et al. 2018; Sayedalamin et al. 2017; Merdad et al. 2017; Elsafi et al. 2017; Al Habeeb et al. 2017; Flayou et al. 2016; Al Bshabshe et al. 2016; Almohsen et al. 2016; Afzal Aghaee et al. 2015; Alsaied et al. 2012). Three studies were conducted in general population (Alam 2007; Shahbazian et al. 2006; El-Shoubaki and Bener 2005). One study was among out-patients in a hospital (Agrawal et al. 2017).

The 14 studies assessed the willingness to donate organ but only 10 studies evaluated the knowledge. Two studies found the association between knowledge and willingness (Merdad et al. 2017; Afzal Aghaee et al. 2015). These studies reveled that improvements in knowledge increased the willingness.

**Fig. 2:**
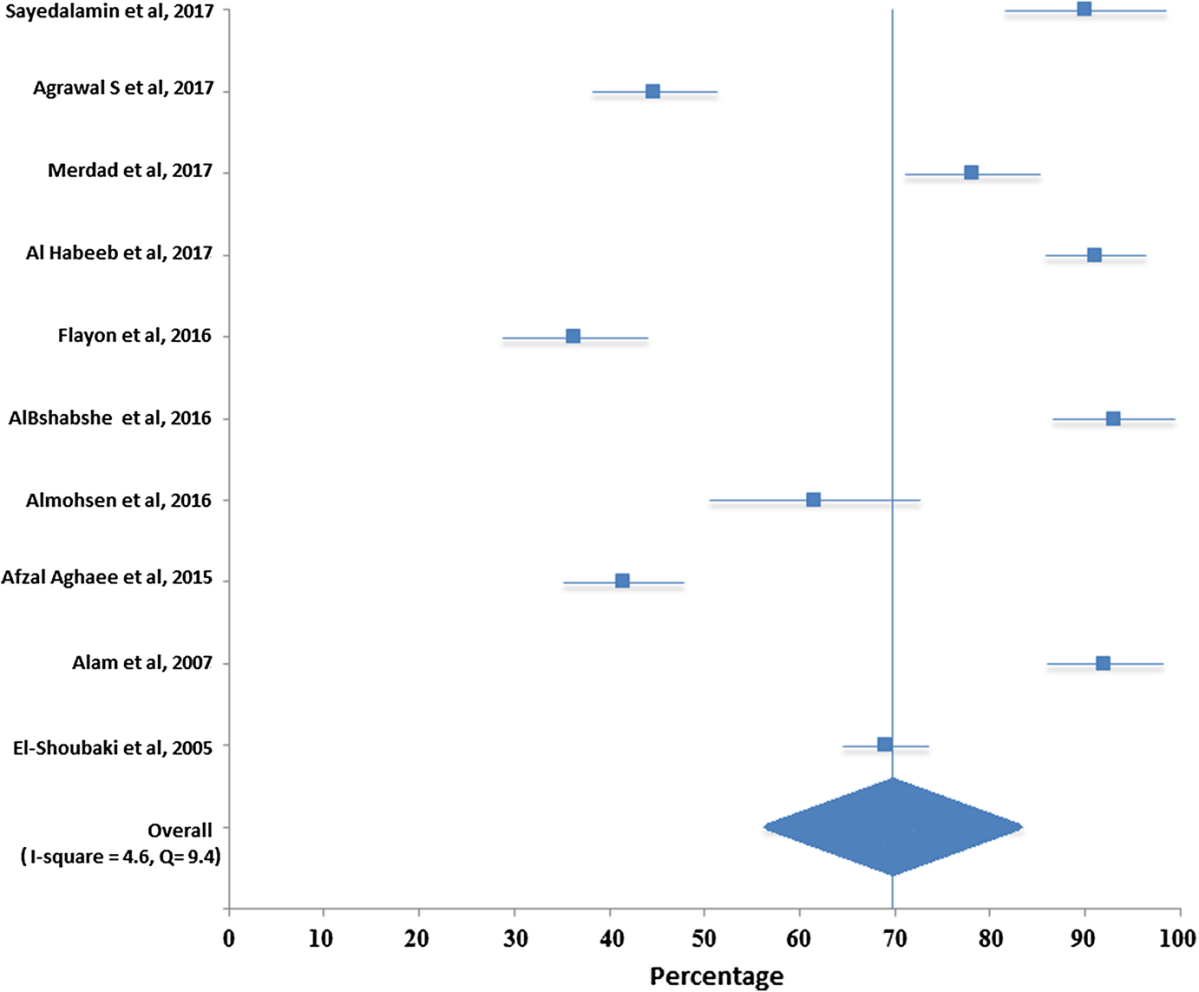
Forest plot for pooled overall knowledge regarding organ donation

**Fig. 3:**
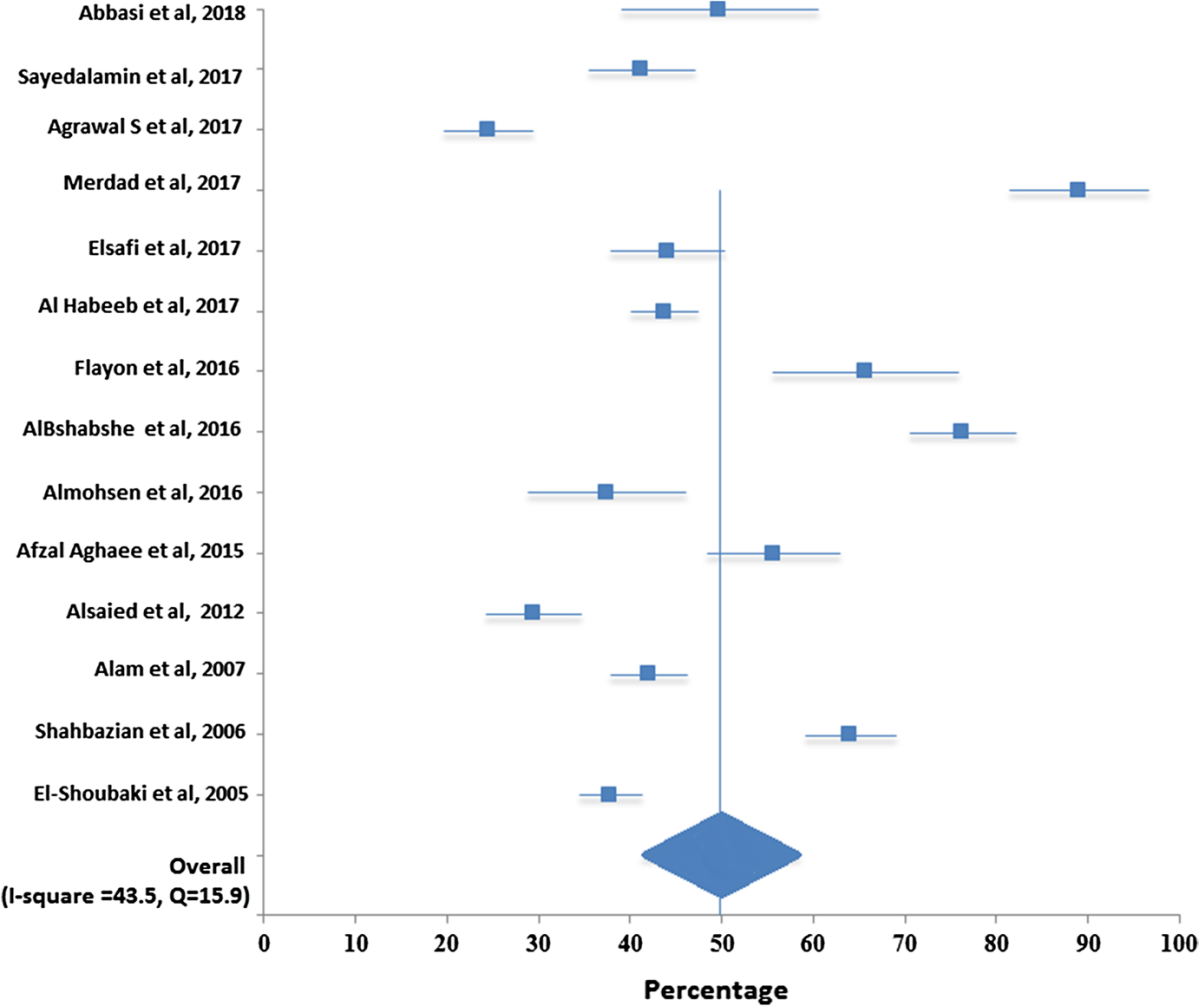
Forest plot for pooled overall willingness regarding organ donation

Total pooled sample size for assessing knowledge was 6697 and for willingness was 8714. Table 2 and Fig. 2 show that the pooled overall knowledge regarding organ donation was 69% with a 95% CI [64.5, 73.5]. Table 3 and Fig. 3 show that the pooled overall willingness to donate organ was 49.8 with a 95% CI [41.3, 58.4].

**Table 2:** Meta-analysis results of knowledge of organ donation in Middle East

Study	Sample size	Percentage	95% confidence interval
Sayedalamin et al. (2017)	481	90.0	(81.5, 98.5)
Agrawal et al. (2017)	403	44.7	(38.1, 51.2)
Merdad et al. (2017)	597	78.1	(71.0, 85.1)
AlHabeeb et al. (2017)	1250	91.0	(85.8, 96.3)
Flayou et al. (2016)	245	36.3	(28.8, 43.9)
Al Bshabshe et al. (2016)	873	93.0	(86.6, 99.4)
Almohsen et al. (2016)	195	61.5	(50.5, 72.5)
Afzal Aghaee et al. (2015)	400	41.5	(35.2, 47.8)
Alam et al. (2007)	948	92.0	(85.9, 98.1)
El-Shoubaki and Bener (2005)	1305	69.0	(64.5, 73.5)

**Table 3:** Meta-analysis results of willingness of organ donation in Middle East

Study	Sample size	Percentage	95% confidence interval
Abbasi et al. (2018)	165	49.7	(38.9, 60.5)
Sayedalamin et al. (2017)	481	41.2	(35.5, 46.9)
Agrawal et al. (2017)	403	24.5	(19.7, 29.3)
Merdad et al. (2017)	597	89.0	(81.4, 96.6)
Elsafi et al. (2017)	434	44.0	(37.8, 50.2)
Al Habeeb et al. (2017)	1250	43.6	(39.9, 47.3)
Flayou et al. (2016)	245	65.7	(55.6, 75.8)
Al Bshabshe et al. (2016)	873	76.2	(70.4, 82.0)
Almohsen et al. (2016)	195	37.4	(28.8, 46.0)
Afzal Aghaee et al. (2015)	400	55.6	(48.3, 62.9
Alsaied et al. (2012)	418	29.3	(24.1, 34.5)
Alam et al. (2007)	948	42.0	(37.9, 46.1)
Shahbazian et al. (2006)	1000	64.0	(59.0, 69.0)
El-Shoubaki and Bener (2005)	1305	37.8	(34.5, 41.1)
Summary	8714	49.8	(41.3, 58.4)

## Discussion

To the best of our knowledge, this is the first attempt to conduct a meta-analysis to evaluate the knowledge and willingness of organ donation in the Middle East. Knowledge of organ donation was found good but the willingness to donate was poor in the Middle East. Studies had demonstrated that willingness to donate organs is related to knowledge about organ donation. Knowledge was measured by administering questionnaires which address general knowledge on organ donation, awareness on procurement and distribution of donated organs, religious and cultural understanding on organ donation (Abbasi et al. 2018; Sayedalamin et al. 2017; Merdad et al. 2017; Elsafi et al. 2017; Al Habeeb et al. 2017; Flayou et al. 2016; Al Bshabshe et al. 2016; Almohsen et al. 2016; Afzal Aghaee et al. 2015; Alsaied et al. 2012).

Religious and cultural reasons for not donating organs in the adult general population ranged from 44.8 to 49.8% (Agrawal et al. 2017; Al Habeeb et al. 2017). In contrast, religious and cultural reasons for not donating organs among healthcare professionals were less when compared to general population; physicians (12.3%), nurses (26.1%) and allied health personnel (36.1%) (Alsaied et al. 2012). Aghaee et al. showed that the willingness to donate organs among students who were aware of religious leader’s opinion was 2.56 times more when compared to those who were not (Afzal Aghaee et al. 2015). This shows the importance of religious belief in organ donation attitude.

Tong et al. conducted a meta-analysis in 2013, by pooling four studies from USA, Canada, and Spain and found that the willingness to donate an organ to an unknown person was 33% with a CI (23, 25) (Tong et al. 2013). On the other hand, knowledge regarding the organ donation was 76.7% with a 95% CI (46.2, 97) (Tong et al. 2013). The present study revealed a better willingness to donate organ in Middle Eastern countries when compared to the Western countries; however, the knowledge about organ donation was poor. Li et al. conducted a meta-analysis to find out the efficacy of community-based interventions in the willingness of organ donation (Li et al. 2015). Subjects received a wide range of community-based interventions had higher levels of willingness to donate organs and had 1.7 times more tendency to commit as an organ donor (Li et al. 2015).

Transplantation activities across the globe and within the world regions vary significantly (Masri and Haberal 2013). The Middle East Society for Organ Transplantation (MESOT) was established in Turkey in 1987 with objectives to enhance and promote education and to facilitate research and collaboration in organ transplantation in the Middle Eastern, North African and near Mid-Asian countries (Masri and Haberal 2013; Shaheen 2009). Qatar is a member state of MESOT where the first kidney transplantation was performed in 1986 and a legislation allowing deceased donation was enacted in 1997 (Martin and Fadhil 2014). However, the organ donation activity was uncommon until the development of Doha Donation Accord (DDA) in 2009. The DDA established a new ethical framework for practice based on WHO guidance and the Declaration of Istanbul on Organ Trafficking and Transplant Tourism (Martin and Fadhil 2014).The first liver transplant was performed then in 2011 (Khalaf et al. 2013). High prevalence of chronic and end-stage diseases raised the demand of transplantable organs; however, shortage of supply of organs still remain crucial (Khalaf et al. 2013; Rashed and Aboud 2004). In addition, issues in donor identification, reporting, diagnosis, management, documentation, and obtaining consent for donation led to underutilization of transplantable organs (Khalaf et al. 2013). The Qatar Center for Organ Transplantation (QCOT) developed aggressive plans to address these problems and to improve the number and quality of available deceased donors.

However, there is a lack of community-based research from Qatar to explore the social and cultural factors influencing organ donation. Qatar has a diverse socioeconomic and multiethnic population and therefore community-based studies are crucial in addressing the social and cultural factors determining ‘willingness to donate’ in different communities. Qatar National Research Fund (QNRF) funded a nationwide cross-sectional survey among the households and qualitative studies among healthcare professionals in Qatar to understand various social and cultural determinants of organ donation as well as the system level issues in the organ donation process.

In Qatar, Al-Thani and colleagues recently conducted an extensive study on factors influencing organ donation (Agarwal et al. 2018). The research project used mixed-method design using qualitative and quantitative methods. The qualitative component of this research project was focus group discussions among the healthcare workers which aimed to understand the factors influencing organ donation among the general population they interact with during their professional life. The results revealed that lack of awareness and information about the process of organ donation acts as main barrier for both deceased and living organ donation (Rashed and Aboud 2004). The quantitative part of the project involved cross-sectional survey among the general population to understand the knowledge, awareness, and practice among them. The survey questionnaire was constructed based on the ‘theory of planned behavior’ and validated in the social and cultural context of Arab world, in Qatar (Singh et al. 2018).

Hospitals in the Middle East countries should have their own donor coordinators, who will interact with family members of the potential donors during the early phases itself to improve the family consent rates. More focus on education programs and advertisement are needed to promote awareness and bring positive changes in public attitudes. The opting out policy in organ donation alone may not be sufficient to improve the donation rates, but intensive and systematized efforts are much needed to prevent further avoidable deaths of patients on organ waiting lists.

## Strength and Limitations

To the best of our knowledge, this study is the first of its kind from the Middle East region. The meta-analysis included a total pooled sample size of 6697 for assessing knowledge and 8714 for willingness. One of the limitations in the study was significant variations in the knowledge and willingness level between the studies; this might be because of the convenient sampling technique employed. However, we used random effect model to address this issue. In addition, most of the questions on knowledge and willingness to donate were yes or no type along with other questions. These questions were very wide and the questionnaires were not validated more often.

## Conclusions

Pooled estimates showed that the knowledge of organ donation was good but the willingness was poor in the Middle Eastern countries. Organ donation and transplantation occurs only if the concerned medical community is competent enough to address the complexity involved in the process. The social frame playing a major role in this process as the decision for donating organ is determined by a person or by group of persons standing within their socioeconomic milieu, cultural pretext, religious biases, and multitude of other demographic and social factors. Factors such as awareness, willingness, and knowledge cutting through different axes of socio-demographic factors; family’s part in making a decision on organ donation; religious, traditional and spiritual believes that evidently interact with decisions; and status of ethnic, minority and immigrant populace, are all influencing the act of organ donation. These factors are also interrelated and are complicated with the part of the world they actually exists. Although the majority of these findings are already incorporated into organizational systems across the world, region-specific studies are more crucial to end up in good policy initiatives supported by sound legislature.

## Electronic supplementary material

Below is the link to the electronic supplementary material.

Supplementary material 1 (DOCX 27 kb)
